# Thirdhand smoke beliefs and behaviors among families of primary school children in Shanghai

**DOI:** 10.18332/tid/132289

**Published:** 2021-02-10

**Authors:** Zhilan Xie, Minzhi Chen, Zhicong Fu, Yunjiang He, Yi Tian, Xiaohong Zhang, Nannan Feng

**Affiliations:** 1 Department of Epidemiology and Biostatistics, School of Public Health, Shanghai Jiao Tong University School of Medicine, Shanghai, China

**Keywords:** primary school children, smoke-free home, thirdhand smoke, smoking behaviors, beliefs about thirdhand smoke scale (BATHS)

## Abstract

**INTRODUCTION:**

There are few reports on the beliefs about thirdhand smoke in Chinese families with primary school children. This study aims to understand the beliefs about thirdhand smoke among parents or grandparents of primary school children in Shanghai and to provide an evidence base to incorporate thirdhand smoke preventative action into tobacco control interventions.

**METHODS:**

We performed a cross-sectional survey among parents and grandparents of children aged 6–13 years in the Changjiang Road Primary School and recruited 843 participants to make assessments on the ‘beliefs about thirdhand smoke’ (BATHS) scale. Sociodemographic details including age, gender, marital status, education level, personal income and type of home ownership (new house, secondhand house with or without redecoration) and health status of children (whether they suffered from respiratory diseases or not) were investigated. Scale assessment, univariate and multivariate analyses to explore the factors influencing the BATHS scale and subscale scores, were performed using SPSS version 22.0.

**RESULTS:**

Participants who were aged >65 years were more likely to get lower scores on the BATHS scale (OR=0.476; 95% CI: 0.311–0.728, p=0.001). Undergraduates (OR=1.190; 95% CI: 1.020–1.388, p=0.027) and graduates (OR=1.4490; 95% CI: 1.102–1.906, p=0.008) obtained higher scores. Moreover, the scores of residents living in a secondhand house with redecoration (OR=0.882; 95% CI: 0.782–0.995, p=0.041) and without redecoration (OR=0.801; 95% CI: 0.698–0.919, p=0.002) were lower compared with those of new-house owners. The scores for participants whose children suffered from respiratory diseases in the past six months (OR=1.104; 95% CI: 1.003–1.216, p=0.043) were higher than those whose children had no respiratory diseases.

**CONCLUSIONS:**

This study shows that younger people, females, those with higher incomes, and higher education levels, were more likely to believe the thirdhand smoke impacts on health and its persistence in the environment. Our findings can guide targeted actions for smoke-free home interventions.

## INTRODUCTION

Exposure to environmental tobacco smoke (ETS) describes any tobacco smoke exposure other than active smoking and comprises secondhand smoke (SHS) and thirdhand smoke (THS)^1^. It was identified as a public health problem in the 1986 US Surgeon General’s Report on the adverse health effects of involuntary smoking^2^. Numerous studies have shown that SHS or ETS exposure has many adverse health consequences^2-5^. Consequently, governments around the world have implemented laws to prohibit smoking in public and work places^3,6^. In addition to reducing cigarette smoking, smoking restrictions in public places and houses protected people from the health risks of SHS exposure and were a powerful stimulus to adopt voluntary smoke-free policies in homes and cars^7,8^. In China, Shanghai was the first city to legally limit indoor smoking in certain public places within the city since March 2010. However, the average daily tobacco consumption in urban areas of Shanghai in 2015 was not significantly different from that in 2010 (14.3±9.0 vs 15.3±28.2 cigarettes/day, p>0.05)^9^. Therefore, SHS or ETS exposure in private places, such as homes, is still an important issue affecting the health of non-smokers.

THS refers to tobacco smoke toxicants that settle on indoor surfaces, fabrics and dust. It lingers for a long time, well after tobacco smoking has taken place^10,11^. It can also be re-emitted into the gas phase and undergo chemical transformations as it reacts with ozone^12^ and nitrous acid^13^ gases that are commonly present in houses^14^ and cars^7,13^. The chemical transformations may yield secondary highly carcinogenic contaminants such as: formaldehyde^15^, tobacco-specific nitrosamines, 4-(methylnitrosamino)-4-(3-pyridyl)butanal, and 4-(methylnitrosamino)-1-(3-pyridyl)-1-butanone^13,16^ as well as tobacco-related toxicants, including volatile N-nitrosamines, aromatic amides, polycyclic aromatic hydrocarbons, and volatile carbonyls^16,17^.

Previous studies have demonstrated the harmful effects of THS in cells, animal models, and people including children^18^. THS collected from smokers’ homes contained high levels of nicotine^19^. THS exposure caused functional alterations and cytotoxicity in both animal and human cells^20^, including mitochondrial stress, dysregulations of gene expression^21^ and DNA damage^20-23^. THS exposure in mice and fetal rats caused changes in liver, lung, skin tissue and behavior^24-27^. Several studies demonstrated that infants and children are at a higher risk of THS exposure than adults because they breathe faster, have thinner skin, and stay longer in homes and on the floor, where dust is deposited, disturbed, and resuspended in the air^18^.

One study found that compared to 65% of non-smokers, only 43% of smokers agreed that THS harms children. Moreover, strict prohibition of smoking in homes was more prevalent among non-smokers^28^. Consequently, parental awareness and beliefs on the impact of THS on children’s health can affect their behavior directly, and determine whether children avoid THS. Therefore, it is necessary to investigate the belief scale of THS among parents. Our study aims to understand the beliefs about THS among parents of primary school children in Shanghai. Our study contributes to promoting smoke-free policies at home.

## METHODS

### Participants and procedures

We performed a cross-sectional survey in Changjiang Road Primary School in June 2019. The Changjiang Road Primary School is located in Songnan town, which is a medium-sized economic area in Baoshan District, Shanghai. There were 885 children in the primary school, half were non-Shanghai residents, which represents the general situation of primary schools in Baoshan District. The paper-based survey questionnaires were distributed to the pupils by their teachers and were taken home to their parents. If both parents smoked or neither parent smoked, either person could fill in the questionnaire. If only one of the parents smoked, we encouraged the person who smoked to fill in the questionnaire. Our aim was to recruit more smokers in order to better understand their perceptions of THS. All participants were required to sign the informed consent form, which contained information such as the purpose of the survey, the content of the study, the inclusion and exclusion criteria, the possible risks and benefits, and privacy protection. It also stated that participants can withdraw from the survey at any time. The questionnaires were returned to the teachers the next day and then mailed to the research team. The information collection and data import and analysis were completed by two independent researchers, who could not contact the participants. The survey was completely anonymous. The study was approved by the Ethics Committee of School of Public Health, Shanghai Jiao Tong University School of Medicine.

In total, we received 843 questionnaires, a response rate of 95.25%. The correlation coefficient in social psychology was about 0.21 according to the review of general psychology studies^29^. Based on that, we used the website ‘Understanding Statistical Power and Significance Testing’ (https://rpsychologist.com/d3/nhst/) to calculate the minimal sample size. The significance level was set as α=0.05, power at 1-β=0.90 and effect size Cohen’s d=0.21. The sample size of 843 met the required minimum of 238.

### Measures

#### Sociodemographics

Participants were asked to indicate age, sex, marital status, education level, personal income and home ownership (new house, secondhand house with or without redecoration).

### The ‘beliefs about thirdhand smoke’ or BATHS scale

We investigated the participants’ beliefs about THS using the ‘beliefs about thirdhand smoke (BATHS)’ scale^30^ (Supplementary file Table S1). We translated the BATHS scale into Chinese and carried out the survey in the Chinese population. The scale assesses the THS persistence in the environment (Factor 1) and THS impact on health (Factor 2). Factor 1 includes items describing THS in the building environment, capturing persistence of smoke particles, accumulation of THS, and ineffectiveness of THS reduction by means other than not smoking in the house. Factor 2 includes health impact of THS and transmission of THS through means other than the air^30^. Participants were asked whether they strongly disagreed, disagreed, not sure, agreed, or strongly agreed, with the statements coded on a scale 1–5.

#### Smoking behaviors

Several questions were asked to the participants: 1) ‘Do you smoke?’. A smoker was defined as someone who has consumed tobacco at least once in the past year; 2) ‘Do you smoke in front of children?’; and 3) ‘How many people smoke in your family?’.

#### Information about children

We also asked the participants several questions about their children: 1) ‘Is your child a boy or a girl?’; 2) ‘How old is he or she?’; and 3) ‘Has your child suffered from a respiratory disease in the past 6 months, including cold, pneumonia, bronchitis, asthma, tracheitis, laryngitis or rhinitis?’.

#### Data analyses plan

We had checked the data and found that <10% of the data were missing. We eliminated rows with missing data when performing the data analyses. Data were verified for normality of distribution and equality of variances by SPSS version 22.0. Descriptive statistics for participant demographics were calculated. The quantitative variables are presented as mean (± SD) and qualitative data are described as frequency and percentage. We performed t-test/ANOVA (normal distribution) or Mann-Whitney U/Kruskal-Wallis test (non-normal distribution) to assess the difference between scale scores by participant characteristics. We then conducted multivariate analysis to explore the factors influencing the BATHS scale and subscale, using the generalized linear model. Independent variables included demographics and variables identified by univariate analysis that had a statistically significant association with the BATHS score. Odds ratios, adjusted for parent gender, parent age, parent education level and family income, were calculated for each dependent variable.

We conducted the exploratory factor analysis to assess the fit of the two-factor solution through principal component analysis (PCA) and calculated Cronbach’s alpha using SPSS 22.0. Significance test was bilateral and the level of statistical significance was set at p<0.05 for all analyses.

## RESULTS

### Characteristics of participants

Demographics of respondents are shown in Table 1. The mean age of the participants was 39.42 (SD=7.46) years. Over half of the participants were male (54.10%). The majority of participants were married (93.59%), aged <40 years (62.28%), had a high school or higher education level (83.21%), and 80.77% reported an average annual income of ≥50000 RMB (100 Chinese Renminbi about 15 US$). Most participants lived in their own new house (65.09%). More than half of their children reported respiratory diseases in the past six months (60.48%). There were 359 smokers, accounting for 42.86% of the participants.

**Table 1:** Characteristics of participants and differences in beliefs about thirdhand smoke (THS) scale and subscale scores among families of primary school children in Shanghai, 2019 (N=843)

*Characteristics*	*n (%)*	*THS health Mean±SD*	*p*	*THS persistence Mean±SD*	*p*	*Overall score Mean±SD*	*p*
**Sex**			**0.001**		0.221		**0.006**
Female	386 (45.90)	4.22±0.69		3.96±0.76		4.10±0.68	
Male	455 (54.10)	4.04±0.75		3.88±0.75		3.97±0.70	
**Relationship status**			0.345		0.336		0.283
Married	788 (93.59)	4.13±0.71		3.92 ±0.74		4.04±0.68	
Single or casually dating	5 (0.59)	4.40±0.95		3.88±0.43		4.17±0.71	
Separated or divorced	42 (4.99)	4.13±0.77		3.79±0.91		3.98±0.76	
Widowed	7 (0.83)	3.60±1.80		3.50±1.91		3.56±1.83	
**Education level**			**<0.001**		**<0.001**		**<0.001**
≤Junior high school	140 (16.79)	3.94±0.80		3.65±0.82		3.81±0.76	
Senior high schools	314 (37.65)	4.05±0.72		3.84±0.75		3.96±0.69	
Undergraduate	348 (41.73)	4.22±0.66		4.05±0.70		4.14±0.65	
≥Master’s	32 (3.84)	4.49±0.86		4.24±0.61		4.38±0.63	
**Average annual income/person** (10000 RMB)			**0.012**		**<0.001**		**0.001**
≤5	158 (19.24)	4.01±0.78		3.73±0.79		3.89±0.72	
>5 and ≤7	186 (22.67)	4.13±0.71		3.90±0.68		4.03±0.65	
>7 and ≤11	259 (31.55)	4.06±0.72		3.86±0.78		3.97±0.71	
>11	218 (26.55)	4.28±0.67		4.12±0.72		4.21±0.66	
**Smoking status**			**<0.001**		**<0.001**		**<0.001**
Smoker	360 (42.86)	4.02±0.71		3.79±0.69		3.91±0.65	
Non-smoker	480 (57.14)	4.20±0.72		4.01±0.79		4.12±0.72	
**House situation**			**0.003**		**<0.001**		**<0.001**
Owned new house	537 (65.09)	4.18±0.71		3.98±0.73		4.09±0.68	
Secondhand house with redecoration	168 (20.36)	4.07±0.71		3.88±0.75		3.98±0.69	
Secondhand house without redecoration	118 (14.30)	3.94±0.78		3.68±0.81		3.83±0.73	
**Number of smokers living together**			**<0.001**		**<0.001**		**<0.001**
0	371 (44.06)	4.22±0.72		4.03±0.77		4.14±0.70	
1	394 (46.79)	4.04±0.72		3.81±0.73		3.94±0.68	
>1	77 (9.14)	4.10±0.73		3.87±0.75		4.00±0.68	
**Age** (years)			**<0.001**		**<0.001**		**<0.001**
<40	525 (62.28)	4.18±0.73		3.97±0.77		4.09±0.71	
40–65	303 (35.94)	4.06±0.68		3.84±0.71		3.96±0.64	
>65	15 (1.78)	3.24±1.04		3.10±0.85		3.18±0.94	
**Health status of children**			**0.031**		**0.009**		**0.006**
No respiratory diseases	332 (39.52)	4.06±0.73		3.83±0.74		3.96±0.68	
Suffered from respiratory diseases	508 (60.48)	4.17±0.72		3.97±0.76		4.08±0.70	

RMB: 100 Chinese Renminbi about 15 US$. SD: standard deviation.

### BATHS scale assessment

The reliability of the 9-item scale measured by Cronbach’s alpha was >0.90 (raw 0.907, standardized 0.912) and the reliability of the subscales was strong (raw/standardized Cronbach’s alpha=0.791/0.807 for Factor 1 THS persistent, 0.877/0.880 for Factor 2 THS health) (Table 2).

**Table 2:** Reliability assessment and factor analysis of beliefs about thirdhand smoke (BATHS) scale among families of primary school children in Shanghai, 2019 (N=843)

*Scale item*	*Mean±SD*	*Factor loadings*	
		*THS health*	*THS persistence*
Breathing air in a room today where people smoked yesterday can harm the health of infants and children.	4.42±0.79	0.687	
Breathing air in a room today where people smoked yesterday can harm the health of adults.	4.27±0.83	0.756	
Particles in rooms where people smoked yesterday can cause cancer.	3.95±0.94	0.723	
After smoking a cigarette, smoke particles on skin, hair and clothing can be passed on to others through touch.	4.07±0.88	0.713	
After touching surfaces where cigarette smoke has settled, particles can enter the body through the skin.	3.83±1.01	0.679	
Smoke particles can remain in a room for days.	4.12±0.84		0.731
Smoke particles can remain in a room for weeks.	3.69±0.99		0.711
Smoke particles get absorbed into furniture and walls.	4.05±0.89		0.631
Opening windows or using air conditioners does not eliminate all smoke particles in a room.	3.72±1.12		0.397

The 9-item scale’s reliability as measured with Cronbach’s alpha was greater than 0.90 (raw 0.907, standardized 0.912) and strong reliability in the subscales (raw/standardized Cronbach’s alpha=0.791/0.807 for Factor 1, and 0.877/0.880 for Factor 2). Factor 1 includes four items related to THS persistence in the environment and Factor 2 includes five items related to THS impact on health.

### Univariate analysis for BATHS scale and subscale

First, we performed univariate analysis for BATHS scale assessment (Table 1). Female (female 4.10±0.68, male 3.97±0.70, p=0.006), younger participants (<40 years 4.09±0.71, 40–65 years 3.96±0.64, >65 years 3.18±0.94, p<0.001), participants with higher education (≤ junior high school 3.81±0.76, senior high school 3.96±0.69, undergraduate 4.14±0.65, ≥ Master’s 4.38±0.63, p<0.001), new-house owners (new house 4.09±0.68, secondhand house with redecoration 3.98±0.69, secondhand house without redecoration 3.83±0.73, p<0.001), and non-smokers (smokers 3.91±0.65, non-smokers 4.12±0.72, p<0.001) were more likely to obtain higher scores in the BATHS scale. The annual income of participants (given per person in units of 10000 RMB) also influenced their BATHS scale scores significantly (≤5: 3.89±0.72; >5 and ≤7: 4.03±0.65; >7 and ≤11: 3.97±0.71; >11: 4.21±0.66; p=0.001). Participants whose children suffered from respiratory diseases in the past six months had higher scores (suffered respiratory diseases 4.08±0.70, otherwise 3.96±0.68, p=0.006). The results also indicated that when more smokers lived together they obtained lower scores in the BATHS scale (p<0.001).

We also performed univariate analysis for the BATHS subscale and found that the results of THS impact were almost the same as that of the BATHS scale (Table 1). However, there was no significant difference by sex in THS persistence in the environment.

### Multivariate analysis for BATHS scale and subscale

We performed multivariable analysis using a generalized linear model to predict the factors influencing the score of the BATHS scale (Table 3). Model included the following variables: sex, age, education level, smoking status, house situation, numbers of smokers living together, health status of children, and annual income. Regarding beliefs about THS, the overall model was significant (p<0.05). The model illustrated that the BATHS scale scores of participants aged >65 years were lower than for participants aged <40 years (OR=0.476; 95% CI: 0.311–0.728, p=0.001). The BATHS scale scores of participants with a Bachelor’s degree (OR=1.190; 95% CI: 1.020–1.388, p=0.027) and Master’s degree or better (OR=1.449; 95% CI: 1.102–1.906, p=0.008) were higher than for those who had junior high school education or lower. In addition, the results indicated that the scores of residents living in a secondhand house with redecoration (OR=0.882; 95% CI: 0.782–0.995, p=0.041) and secondhand house without redecoration (OR=0.801; 95% CI: 0.698–0.919, p=0.002) were lower compared with those of new-house owners. The results also showed that the scores for participants whose children suffered respiratory diseases in the past six months (OR=1.104; 95% CI: 1.003–1.216, p=0.043) were higher than those whose children had no respiratory diseases.

**Table 3:** Analysis the factors influencing the score of beliefs about thirdhand smoke (BATHS) using generalized linear model among families of primary school children in Shanghai, 2019 (N=843)

*Variable*	*Categories*	*β*	*Wald χ^2^*	OR*	*95% CI*	*p*
**Sex**	Male			1		
	Female	0.052	0.876	1.053	0.945–1.175	0.349
**Age** (years)	<40			1		
	40–65	-0.081	2.456	0.922	0.833–1.021	0.117
	>65	-0.743	11.714	0.476	0.311–0.728	**0.001**
**Education level**	≤Junior high school			1		
	Senior high school	0.042	-0.306	1.042	1.102–1.906	0.580
	Undergraduate	0.174	4.902	1.190	1.020–1.388	**0.027**
	≥Master’s	0.371	7.030	1.449	1.102–1.906	**0.008**
**Average annual income/person** (10000 RMB)	≤5		1			
	>5 and ≤7	0.094	1.521	1.098	0.946–1.274	0.217
	>7 and ≤11	-0.027	0.140	0.973	0.843–1.123	0.708
	>11	0.135	2.982	1.145	0.982–1.335	0.084
**Smoking status**	Smoker			1		
	Non-smoker	0.057	0.523	1.509	0.907–1.236	0.470
**House situation**	Owned new house			1		
	Secondhand house with redecoration	-0.126	4.187	0.882	0.782–0.995	**0.041**
	Secondhand house without redecoration	-0.222	9.987	0.801	0.698–0.919	**0.002**
**Number of smokers living together**	0			1		
	1	-0.110	2.312	0.896	0.778–1.032	0.128
	>1	-0.036	0.123	0.964	0.787–1.181	0.725
**Health status of child**	No respiratory diseases			1		
	Suffered from respiratory diseases	0.099	4.088	1.104	1.003–1.216	**0.043**

* OR for BATHS score was adjusted for sex, age, education level, annual income, smoking status, house situation, the number of smokers living together, and health status of child. RMB: 100 Chinese Renminbi about 15 US$.

Multivariable analysis for environmental persistence factor of THS in the BATHS subscale (Table 4) revealed that average scores of persons aged >65 years were lower than those aged <40 years (OR=0.506; 95% CI: 0.319–0.801, p=0.004). Scores of THS persistence were increased by 23.1% and 44.7%, respectively, in participants with college education, or Master’s or better, in comparison with those with junior high school education (undergraduate OR=1.231; 95% CI: 1.044–1.453, p=0.014, and ≥Master’s OR=1.447; 95% CI: 1.077–1.945, p=0.014). Simultaneously, scores assessing THS persistence in participants with average annual income >110000 RMB were higher than those with ≤50000 RMB (OR=1.199; 95% CI: 1.017–1.414, p=0.031). Participants living in a secondhand house without redecoration obtained lower average THS health scores than those of a redecorated house (OR=0.786; 95% CI: 0.678–0.912, p=0.002). The model also showed that average persistence in the environment scores for respondents whose children suffered from respiratory diseases in the past 6 months were higher than those whose children did not (OR=1.124; 95% CI: 1.013–1.246, p=0.028).

**Table 4:** Analysis of the factors influencing the beliefs about thirdhand smoke (BATHS) subscale score of persistence using generalized linear model among families of primary school children in Shanghai, 2019 (N=843)

*Variable*	*Categories*	*β*	*Wald χ^2^*	OR*	*95% CI*	*p*
**Sex**	Male			1		
	Female	-0.031	0.267	0.969	0.862–1.091	0.606
**Age** (years)	<40			1		
	40–65	-0.093	2.793	0.911	0.816–1.016	0.095
	>65	-0.682	8.446	0.506	0.319–0.801	**0.004**
**Education level**	≤Junior high school			1		
	Senior high school	0.068	0.707	1.070	0.914–1.253	0.400
	Undergraduate	0.208	6.082	1.231	1.044–1.453	**0.014**
	≥Master’s	0.370	6.000	1.447	1.077–1.945	**0.014**
**Average annual income/person** (10000 RMB)	≤5		1			
	>5 and ≤7	0.116	2.022	1.123	0.957–1.318	0.155
	>7 and ≤11	0.006	0.005	1.006	0.862–1.173	0.944
	>11	0.182	4.662	1.199	1.017–1.414	**0.031**
**Smoking status**	Smoker			1		
	Non-smoker	0.117	1.874	1.124	0.951–1.328	0.171
**House situation**	Owned new house			1		
	Secondhand house with redecoration	-0.124	3.561	0.883	0.776–1.005	0.059
	Secondhand house without redecoration	-0.240	10.069	0.786	0.678–0.912	**0.002**
**Number of smokers living together**	0			1		
	1	-0.100	1.653	0.905	0.777–1.054	0.199
	>1	-0.023	0.042	0.977	0.785–1.216	0.837
**Health status of child**	No respiratory diseases			1		
	Suffered from respiratory diseases	0.116	4.846	1.124	1.013–1.246	**0.028**

* OR for BATHS subscale score of persistence was adjusted for sex, age, education level, annual income, smoking status, house situation, the number of smokers living together, and health status of child. RMB: 100 Chinese Renminbi about 15 US$.

Generalized linear model evaluating the THS impact on the health factor in the BATHS subscale (Table 5) indicated that the average THS health scores in women were higher than those in men (OR=1.125; 95% CI: 1.004–1.260, p=0.042). Scores of the older people were lower than those of younger people (OR=0.453; 95% CI: 0.290–0.706, p<0.001). Participants with a Master’s, or higher, education level obtained higher scores than those with junior high school education or below (OR=1.445; 95% CI: 1.089–1.918, p=0.011). Participants living in a secondhand house without redecoration had lower scores than owners of a redecorated house (OR=0.817; 95 % CI: 0.807–0.943, p =0.006).

**Table 5:** Analysis of the factors influencing the beliefs about thirdhand smoke (BATHS) subscale score of health using generalized linear model among the families of primary school children in Shanghai, 2019 (N=843)

*Variable*	*Categories*	*β*	*Wald χ^2^*	OR*	*95% CI*	*p*
**Sex**	Male			1		
	Female	0.118	4.125	1.125	1.004–1.260	**0.042**
**Age** (years)	<40			1		
	40–65	-0.072	1.768	0.931	0.837–1.035	0.184
	>65	-0.793	12.198	0.453	0.290–0.706	**<0.001**
**Education level**	≤Junior high school			1		
	Senior high school	0.028	0.126	1.028	0.882–1.198	0.723
	Undergraduate	0.154	3.557	1.167	0.994–1.369	0.059
	≥Master’s	0.368	6.498	1.445	1.089–1.918	**0.011**
**Average annual income/person** (10000 RMB)	≤5			1		
	>5 and ≤7	0.078	0.966	1.081	0.926–1.261	0.326
	>7 and ≤11	-0.058	0.577	0.944	0.813–1.096	0.447
	>11	0.092	1.283	1.097	0.935–1.287	0.257
**Smoking status**	Smoker			1		
	Non-smoker	0.008	0.009	1.008	0.858–1.184	0.925
**House situation**	Owned new house			1		
	Secondhand house with redecoration	-0.202	3.773	0.883	0.779–1.001	0.052
	Secondhand house without redecoration	-0.124	7.651	0.817	0.708–0.943	**0.006**
**Number of smokers living together**	0			1		
	1	-0.114	2.283	0.893	0.770–1.034	0.131
	>1	-0.044	0.167	0.957	0.774–1.183	0.683
**Health status of child**	No respiratory diseases			1		
	Suffered from respiratory diseases	0.086	2.854	1.090	0.986–1.205	0.091

* OR for BATHS subscale score of health was adjusted for sex, age, education level, annual income, smoking status, house situation, the number of smokers living together and health status of child. RMB: 100 Chinese Renminbi about 15 US$.

## DISCUSSION

We investigated the beliefs about THS among parents or grandparents of primary school children in Shanghai in order to provide an evidence base for incorporation of THS actions into tobacco control interventions, in the hope of promoting smoke-free homes. This study indicates that younger people and those who received higher education were more likely to believe that THS would persist in the environment and impact children’ s health, as reported in previous studies^31,32^. Moreover, it is interesting that people who lived in a new house, compared to those living in rented houses without redecoration, were more likely to believe that THS can persist in the environment and influence children’s health. We also found that participants whose children suffered from respiratory diseases believed that THS can persist in the environment for a long time. However, they were not sure about the health impact of THS. A similar result was observed in the high-income group. Our analysis showed that females were more likely to believe THS impacts the health of their children but not the environmental persistence of THS. The BATHS scale scores were not different between smokers and non-smokers, which was inconsistent with another study that indicated that both current and former smokers disagreed with the adverse impacts of THS on children’s health^33^.

### Strengths and limitations

Our study involved a large number of participants to produce robust results. However, there are several limitations. First, this study was carried out in one primary school in Baoshan District, Shanghai, for convenience. The survey was conducted among families and the smokers in the family were encouraged to take part in the questionnaire, therefore the percentage of smokers in our study does not reflect the national rates. Second, we found that the proportion of men (54.1%) in our study was higher than the national (51.3%), according to the sixth national census^34^. This could partially explain why the smoking rate in our study was higher compared with the national. Third, cross-sectional data precludes the inference of causality. It is unclear whether these findings could be adapted to other geographical areas or to adults without school-age children. Additionally, the reliance on parent self-report could lead to response biases.

### Considerations for the future

In 2007, the Framework Convention on Tobacco Control required a total ban on smoking in public places, including all indoor public places, indoor workplaces, public transport and other outdoor areas in China. However, smoking still occurs in many households. Even in the absence of children, smoking can be harmful because toxic contaminants, generated by smoking, can settle on the surfaces of furniture, on skin, hair and the clothing of family members^11-13^. Smoke-free homes are defined as homes where no one is allowed to smoke inside, but smoke-free policies in multi-unit housing do not force smokers to use smoke-free facilities, they simply prevent smokers from smoking in settings where SHS affects others through infiltration^35^. Previous research has indicated that as much as 60% of airflow in multi-unit housing facilities can come from other units^36^. Therefore, how to carry out a smoke ban in families, exploring family THS exposure, intervening in smoking by family members, and promoting infants’ health by reducing smoke exposure, are vital to tobacco control programs. Our findings provide details and reflections for future improvement and implementation of tobacco control programs.

## CONCLUSIONS

Our study shows that older people, males, low-income groups, less educated men, and those who rent houses are less aware of the adverse impacts of THS. Through understanding the *status quo* of THS beliefs among family members, targeted education can be carried on the risks of THS and family members can be encouraged to change their smoking behavior. These actions will help to establish healthy concepts, reduce the harm of tobacco, and eventually help to achieve a smoke-free family environment.
