# Supplementary material for: Thirdhand smoke beliefs and behaviors among families of primary school children in Shanghai
Source: Tob Induc Dis. 2021 Feb 10;19:10. doi: 10.18332/tid/132289 (PMC7873816; doi:10.18332/tid/132289)
Supplement: Supplementary file 1 [file TID-19-10-s1.pdf]

Table S1. Items of the Beliefs about Thirdhand Smoke (BATHS) scale [36]

Scale item	Strongly disagree	Disagree	Not sure	Agree	Strongly agree
Breathing air in a room today where people smoked yesterday can harm the health of infants and children	1	2	3	4	5
Breathing air in a room today where people smoked yesterday can harm the health of adults	1	2	3	4	5
Particles in rooms where people smoked yesterday can cause cancer	1	2	3	4	5
Smoke particles can remain in a room for days.	1	2	3	4	5
Smoke particles can remain in a room for weeks.	1	2	3	4	5
Smoke particles get absorbed into furniture and walls.	1	2	3	4	5
After smoking a cigarette, smoke particles on skin, hair, and clothing can be passed on to others through touch.	1	2	3	4	5
After touching surfaces where cigarette smoke has settled, particles can enter the body through the skin.	1	2	3	4	5
Opening windows or using air conditioners does not eliminate all smoke particles in a room.	1	2	3	4	5

© 2021 Xie Z. et al.
